# Stevens-Johnson Syndrome in a Patient of Color: A Case Report and an Assessment of Diversity in Medical Education Resources

**DOI:** 10.7759/cureus.22245

**Published:** 2022-02-15

**Authors:** Darlene Diep, Bineetha Aluri, Alison Crane, Kathleen Miao, Kamilah S Kannan, Robert Goldsteen

**Affiliations:** 1 Medicine, Burrell College of Osteopathic Medicine, Las Cruces, USA; 2 Medicine, University of Southern California, Los Angeles, USA; 3 Biological Sciences, Wheeler Magnet School, Marietta, USA

**Keywords:** stevens-johnson, skin of color, patient of color, medical training, medical education, fitzpatrick, equity, diversity, carbamazepine

## Abstract

This is a review of a patient encounter that underscores the common trend of insufficient inclusivity and lack of diversity regarding skin of color representation in teaching materials including textbooks in the medical education setup. A Black woman who was treated with carbamazepine for trigeminal neuralgia after a dental procedure presented with upper airway breathing difficulties and facial pain and swelling. After doubling her dose of carbamazepine as advised by her primary care physician, her symptoms continued to worsen, and she was treated in the emergency department for a presumed allergic reaction of unknown etiology. Two days later, her symptoms progressively worsened. She self-admitted to the emergency department, where she required cardiopulmonary resuscitation. Eventually, the formal diagnosis of carbamazepine-induced Stevens-Johnson syndrome (SJS) was made based on history, clinical presentation, and skin biopsy.

The nature of the disease progression in this case prompted our investigation into the lack of representation of skin of color in current medical training resources regarding SJS. Our assessment demonstrates that there is a significant underrepresentation of SJS in skin of color in medical educational resources. Increased inclusivity of skin disorders in patients of color is crucial in training healthcare professionals to recognize life-threatening cutaneous disorders quickly and accurately in such patients.

## Introduction

Stevens-Johnson syndrome (SJS) is a life-threatening immune-mediated hypersensitivity reaction in both the skin and mucosal membranes. SJS is typically caused by an adverse drug reaction from certain medications. It classically presents as atypical targetoid macules that spread centrifugally, ultimately leading to dusky plaques followed by full-thickness skin sloughing; however, it can also present as macular, erythematous, targetoid, or flaccid blistering skin lesions [1].

We describe a case of progression of SJS following carbamazepine use in a female patient of color. The initial presentation included periorbital edema, conjunctivitis, and erythematous atypical targetoid lesions throughout her body for which she sought treatment through various channels. Multiple misdiagnoses and the eventual exacerbation of the patient’s condition culminated in her hospital admission, where she collapsed and was resuscitated. Eventually, the formal diagnosis of carbamazepine-induced SJS was confirmed by biopsy.

The Fitzpatrick skin phototype scale classifies different skin types based on their response to ultraviolet radiation exposure. Fitzpatrick I-III describes “white skin” that easily burns with ultraviolet radiation exposure, while Fitzpatrick IV-VI represents “skin of color”, which readily tans and minimally burns with ultraviolet radiation exposure [2].

Many medical textbooks and web-based resources used by medical professionals and students provide photographs of the cutaneous morphology in SJS. While these photos can be helpful in learning the signs of the reaction, most of the images are of patients of lighter skin tones. Having fewer examples of darker skin tones in education materials can further augment the prevailing inequality in the diagnosis of a life-threatening disease between patients of different skin tones, which disproportionately affects people with skin of color. When medical professionals are disproportionately exposed to one type of skin tone, it may foster an inability to recognize cutaneous symptoms in other types of skin tone, which can delay appropriate and timely care in such patients.

## Case presentation

**Figure 1:**
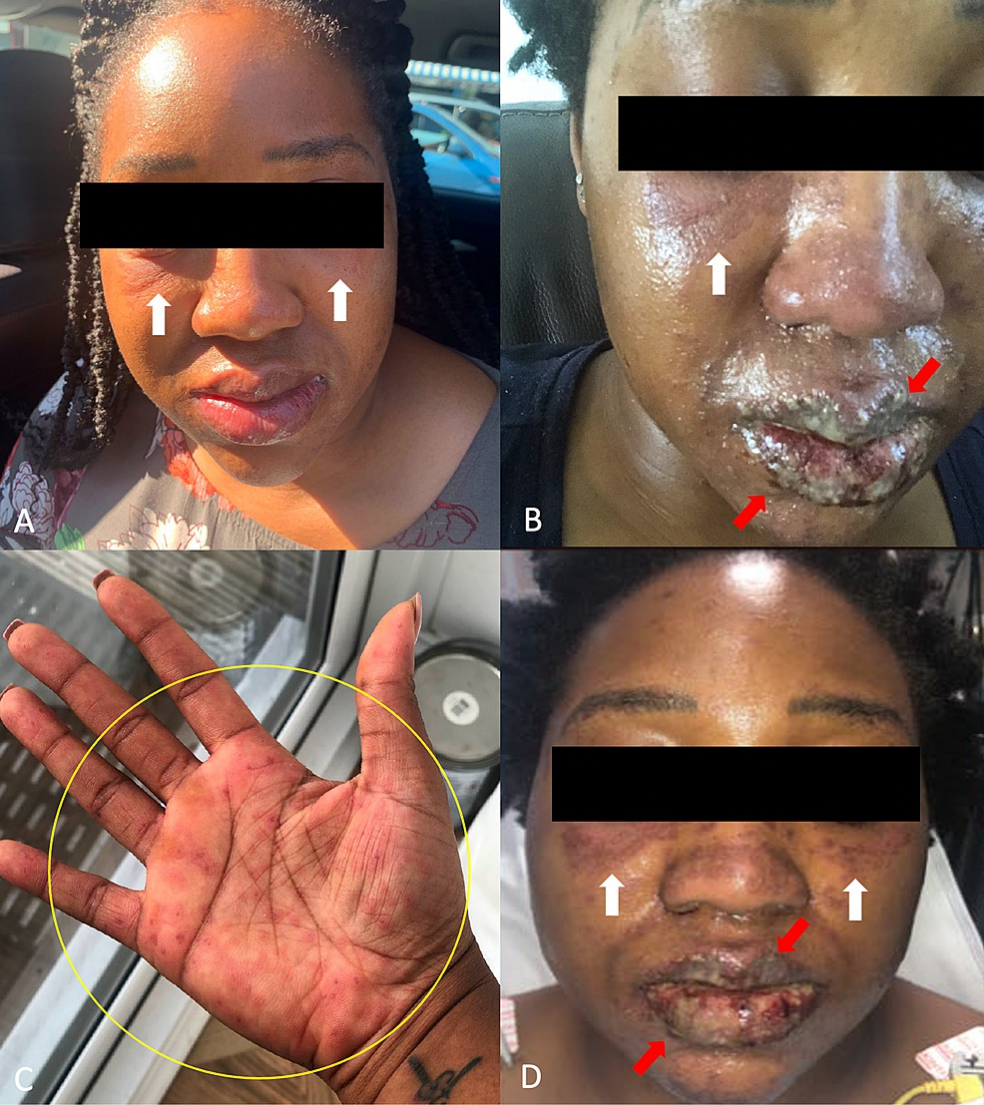
Clinical presentation of Stevens-Johnson syndrome progression From the first day of symptoms (A) to the fifth (B and C) and ninth (D), edema of the eyes (white arrows) and desquamation of the lips (red arrows) developed in a 25-year-old Black woman. Atypical targetoid macules on the palm (yellow circle) were also noted in addition to the extremities, back, and soles of the feet

A 25-year-old Black woman, with Fitzpatrick skin type V (brown skin that rarely burns and tans very easily), presented for the evaluation of small bumps on the tongue and periorbital scaling of six days' duration (Figure 1A). Prior to her visit, she had presented to her primary care for suspected trigeminal neuralgia following a recent dental procedure, for which she had been prescribed carbamazepine and codeine/paracetamol. Her symptoms included facial swelling, dyspnea, and pain. The patient’s primary care physician had recommended doubling the dose of carbamazepine and codeine/paracetamol for pain management.

Thirteen days after doubling the dose, her symptoms had continued to progress with dysphagia, eye discharge, blurred vision, and atypical targetoid macules throughout her body (Figures 1B, 1C). She presented to the emergency room and was given the diagnosis of tonsillitis and conjunctivitis. The emergency medicine physician prescribed an antibiotic (phenoxymethylpenicillin 250 mg), antihistamine (chlorpheniramine 4 mg), and high-potency corticosteroid (prednisolone 15 mg/5 ml). The patient followed this regimen for two days without improvement.

Two days following her emergency room visit, the patient contacted paramedic services for worsening shortness of breath and difficulty swallowing. Examination demonstrated blanching rashes on her palms, soles, extremities, and back. She also developed scaling in her scalp. The paramedics recommended paracetamol. Later that day, the patient self-admitted to the hospital, where she collapsed and was resuscitated. A chest X-ray was taken but was unremarkable.

The patient had a biopsy taken by a dermatologist, which revealed SJS secondary to a carbamazepine-induced drug eruption. Although erythema multiforme major was considered in the differential diagnosis, it was ultimately ruled out since she did not have a history of herpes simplex virus. Her oral involvement advanced to mucosal blistering and ulceration (Figure 1D). Her scalp began to burn. Her vision diminished to 30% of its functioning capacity and she continued to experience keratoconjunctivitis and periorbital edema.

The patient was treated with eye lubricants, topical steroids, prednisolone taper (40 mg once a day, 35 mg once a day for five days, and reduced to 5 mg every five days), topical non-steroidal anti-inflammatory drug (NSAID) gel, and gastrointestinal prophylactic therapy. During her treatment, she experienced hand dermatitis, weight gain, and difficulty sleeping due to steroid use. Her symptoms resolved after a period of mucosal shedding. The examination after the completion of her treatment showed post-inflammatory hyperpigmentation, mucosal scarring, and pharmacophobia. To date, there has been no recurrence.

## Discussion

SJS is an immune-complex-mediated hypersensitivity reaction of the cutaneous and mucosal membranes due to adverse drug reactions. SJS initially appears as a fever, soon followed by cough, conjunctivitis, sore throat, and maculopapular rash that typically appears on the face, chest, and upper trunk. Eventually, this progresses to vesicles appearing on mucosal surfaces such as the eyes, mouth, airway, and genital region. Ulcers caused by SJS can result in odynophagia and dysphagia in patients, causing difficulty with eating or drinking. Healing of damaged skin can lead to hyper- or hypopigmented skin, as well as irreversible mucosal scarring. The effect on mucosal surfaces can also lead to vision loss and tooth decay [1].

Throughout her disease course, our patient experienced a variety of symptoms that could have been investigated by way of several differential diagnoses (Table 1) [3-16]. The morphology of cutaneous lesions and systemic manifestations of SJS can be variable. In 2020, Dutt et al. discussed another case of SJS in a 46-year-old African American female patient [17]. The patient was evaluated at the emergency department three times for suspected upper respiratory tract infection, erythematous macules and papules scattered throughout the body, conjunctival injection, and pain in the hands and feet. She was only admitted on the third visit and required the consultation of numerous specialists before the diagnosis of SJS was established. The similarities to our patient’s case underscore a trend in terms of the lack of early recognition of this life-threatening condition, especially in patients of color.

**Table 1:** Clinical differential diagnoses of Stevens-Johnson syndrome DRESS: drug rash with eosinophilia and systemic

Morphology	Conditions	References
Blisters	Erosive lichen planus	[3]
	Necrolytic acral erythema	[4]
	Pemphigus vulgaris	[5]
	Staphylococcal scalded skin syndrome	[6]
Edematous	Allergic dermatitis	[7]
	Angioedema	[8]
	Orofacial granulomatosis	[9]
	Rosacea	[10]
Macular	DRESS syndrome	[11]
	Erythroderma	[12]
	Secondary syphilis	[13]
Targetoid	Erythema annulare centrifugum	[14]
	Erythema multiforme	[15]
	Lyme disease	[16]

In 2016, Lu et al. observed that Asians (27%) and Blacks (26%) accounted for a disproportionately high number of patients hospitalized with SJS, while White patients (29%) were underrepresented in the data set. They compared the number of hospitalized cases for each race to the U.S. Census data (5% Asian, 12% Black, 67% White) [18]. The higher incidence of hospitalization for SJS observed among skin of color patients emphasizes the importance of early recognition of symptoms in such patients to ensure that treatment is implemented in a timely manner.

However, delayed recognition of visual symptoms is potentially a consequence of deficiencies in medical education and training. Because medical texts tend to prefer images of people with lighter skin tones, students and clinicians have diminished exposure to cutaneous manifestations in patients with skin of color. The underrepresentation of skin of color in medical texts can therefore increase the gap of disparities in health outcomes between patients of light and dark skin tones, as seen in our patient [19]. 

Bellicoso et al. examined a dermatology curriculum at the University of Toronto for inclusivity of skin of color images. The curriculum audit revealed that there were less than 7% of images representing skin of color patients. The downstream effects of the lack of representation were consequently demonstrated in that students were significantly less confident in diagnosing conditions in people with skin of color [20]. 

**Figure 2:**
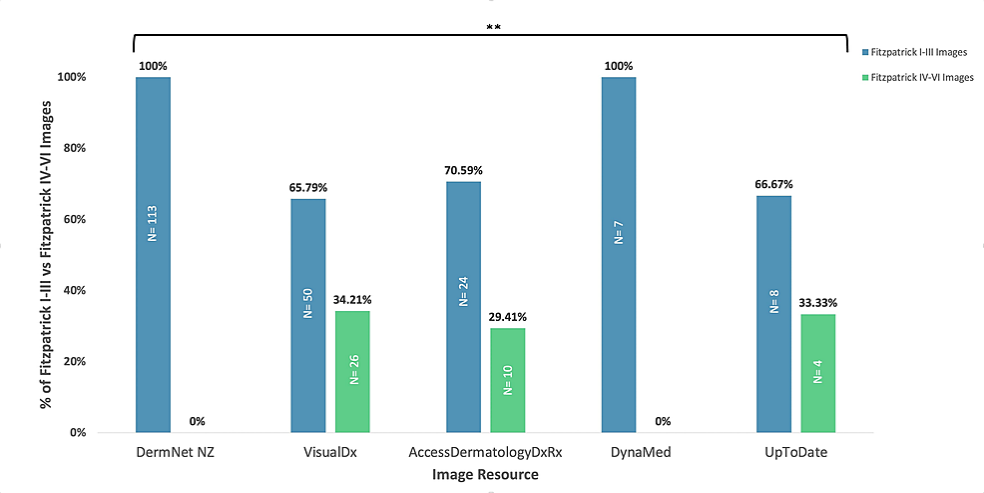
Total percentage of Fitzpatrick I-III vs. Fitzpatrick IV-VI images of SJS in medical education resources **Denotes a very significant difference between the representation of Fitzpatrick I-III skin types and Fitzpatrick IV-VI skin types. A p-value of less than 0.01 was considered statistically very significant SJS: Stevens-Johnson syndrome

Five commonly used web-based resources were selected for a review of images representing Stevens-Johnson syndrome: DermNetNZ, VisualDX, AccessDermatologyDxRx, DynaMed, and UptoDate (Figure 2). In each database, the images were categorized as Fitzpatrick I-III (lighter skin) images or Fitzpatrick IV-VI (darker skin) images.

Among the five resources, VisualDx had the greatest representation of Fitzpatrick IV-VI skin images of SJS, with 26 of the total 76 SJS images (34.21%). AccessDermatologyDxRx and UpToDate had similar rates of representation, with 10 out of 34 Fitzpatrick IV-VI skin SJS images on AccessDermatologyDxRx (29.41%) and four out of 12 Fitzpatrick IV-VI SJS images on UptoDate (33.33%). DermNetNZ did not display any Fitzpatrick IV-VI SJS images even though it had the highest number of SJS images. DynaMed displayed the lowest number of SJS images (n=7), with no Fitzpatrick IV-VI representation. The relationship between the percentages of Fitzpatrick I-III images and Fitzpatrick IV-VI images in medical text resources was analyzed using an unpaired t-test. The representation of Fitzpatrick I-III images was significantly greater than that of Fitzpatrick IV-VI images (p=0.0006).

**Figure 3:**
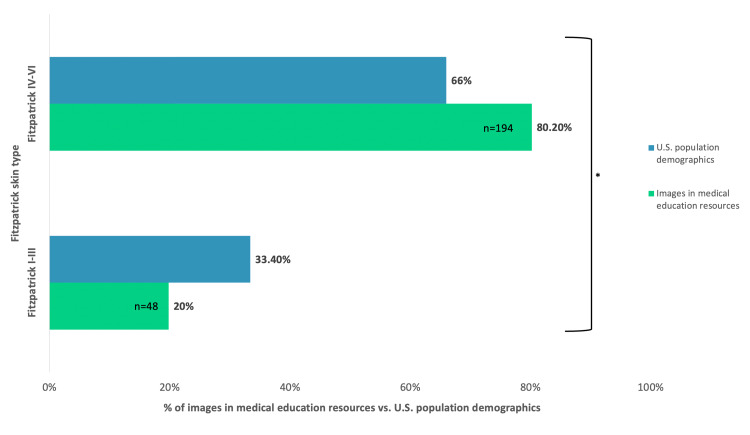
Percentage of Fitzpatrick skin type representation in medical education resources compared to the U.S. Census population demographics *Denotes a significant difference between the representation of images in medical texts compared to the population in the U.S.* *A p-value of less than 0.05 was considered statistically significant

Of the 242 images across all the resources we reviewed, 80.2% were Fitzpatrick I-III images and 19.8% were Fitzpatrick IV-VI. The 2021 U.S. Census report's population estimates for race and origin have projected a rate of 66.0% for White (non-Hispanic or Latino) and Asian individuals. The combined population estimates for Black or African American, Native American and Alaska Native, and Hispanic or Latino were 33.4%. Using the U.S. Census to project the expected percentage of Fitzpatrick IV-VI SJS images, our data indicate that medical education resources do not provide an adequate representation of the various populations in the United States. Although skin tone and Fitzpatrick classification can vary among races, the chi-square test performed to compare observed percentages of medical resource images and expected percentages based on the U.S. Census demonstrated a significant difference (p=0.026) (Figure 3).

The observed percentage of Fitzpatrick IV-VI SJS images in medical education resources is not consistent with the incidence and prevalence of this condition among different races in the population. Overall, there is a disproportionately lower representation when comparing the proportion of Fitzpatrick IV-VI images of SJS to epidemiologic data.

## Conclusions

Stevens-Johnson syndrome can present as a severe cutaneous reaction to medications, such as carbamazepine, within the first month of treatment. Initial symptoms include diffuse erythema, blisters, shortness of breath, and conjunctivitis. Rapid diagnosis and treatment of SJS are critical, to avoid its progress to shock and multiple organ failure.

This case highlights disparities in medical education of cutaneous diseases, specifically SJS, in terms of the representation of skin of color patients in the resources provided for learning. Inability to quickly recognize visual symptoms can lead to worse clinical outcomes and suboptimal care for darker-skin populations due to delays in diagnosis. Our analysis shows an exceedingly low representation of Fitzpatrick IV-VI SJS images in medical educational resources, confirming the discrepancies in medical education. To improve patient care related to preventable life-threatening conditions, educational materials must include a spectrum of skin tones in terms of illustrating pathologies in patients.
